# P01.40. A new approach for quantifying chemosensitizing effects from herb-drug combinations: assessment of Tripterygium Wilfordii-Docetaxel in prostate cancer

**DOI:** 10.1186/1472-6882-12-S1-P40

**Published:** 2012-06-12

**Authors:** Z Wang, S Yeung, T Tran, Y Huang, M Chow

**Affiliations:** 1 CADRE, College of Pharmacy, Western University of Health Sciences, Pomona, USA

## Purpose

Resistance to cancer chemotherapy is a major problem. Herb-drug combinations can offer a new potential to overcome such resistance. To quantitatively assess chemosensitizing effects of herb-drug combinations (HDC), a new approach that takes into account both the chemosensitizing effect (CE) and “safety” considerations of the HDC is proposed and used in describing the cytotoxic activity of Tripterygium wilfordii-docetaxel (TW-Dtx) combination on prostate cancer cell lines.

## Methods

The effect of two extracts of TW with Dtx on Dtx resistant PC3 and DU145 cell lines were compared. Cell viability (cytotoxicity) was determined using sulforhodamine B assay after incubation of the cell line. The IC_50_ of herb (H), drug (D) alone and in combination (IC_50_H, IC_50_D, and IC_50_CD respectively) in resistant cells were obtained. CE and chemosensitizing utility index (CUI) were calculated as: CE = IC_50_D IC_50_CD; CUI=CE (IC_50_H/Con_H_) whereas Con_H_ is the H concentration in the combination.

## Results

 The values for CE (fold change), CUI (fold change), and IC_50_CD (nM) from TW extract A-Dtx treatment in resistant PC3 cells were 3.8 , 5.8, and 5.8, respectively versus 17.1, 22.0, and 1.5, respectively from TW extract B-Dtx treatment when low ConH relative to IC_50_H was used. The corresponding values from extract A versus B-Dtx treatment in resistant DU145 cells were >1.3, >4.5 and 75.3 versus >27.6, >55.2 and 3.63, respectively. However, CE values can dramatically increase with higher ConH.

## Conclusion

Based on the above CE, CUI or ICCD values, TW extract B-Dtx appeared to be consistently superior to extract A-Dtx combination. However, assessment based on CE value alone may be misleading since it can change dramatically with ConH used. CUI together with IC_50_CD are preferred and may prove to be a useful practical tool for assessing chemosensitizing effect of other HDCs.

